# Role of Neurosurgical Interventions in the Treatment of Movement Disorders Like Parkinson’s Disease, Dystonia, and Tourette Syndrome

**DOI:** 10.7759/cureus.72613

**Published:** 2024-10-29

**Authors:** Rachel Ranjan, Anishka Chourey, Yasmin Kabir, Héctor Daniel García Mata, Erika Tiepolo, Ivana Lizeth Fiallos Vinueza, Cara Mohammed, Saacha F Mohammed, Abrar Ahmed Thottakurichi

**Affiliations:** 1 Neurology, St. John’s Medical College, Bangalore, IND; 2 Medicine and Surgery, District Hospital, Karwi, IND; 3 Medicine, Royal College of Surgeons, Manama, BHR; 4 Medicine, National Autonomous University of Mexico, Mexico City, MEX; 5 Neurosurgery, Humanitas, Milan, ITA; 6 Medicine, Università Vita Salute San Raffaele, Milan, ITA; 7 Orthopaedic Surgery, Sangre Grande Hospital, Sangre Grande, TTO; 8 Medicine, Trinity College, University of Dublin, Dublin, IRL; 9 Medicine, Ivane Javakhishvili Tbilisi State University, Tbilisi, GEO

**Keywords:** clinical implications, clinical trials, deep brain stimulation, dystonia, future scope, movement disorders, neuro-ablative therapy, neuromodulation, neurosurgical interventions, tourette syndrome

## Abstract

This article provides an overview of neurosurgical therapies for movement disorders (MDs), including Tourette syndrome, dystonia, Parkinson's disease (PD), and others. It focuses on the benefits of these treatments and suggests directions for further research. A total of 10 years' worth of English-language PubMed articles were combed through, with an emphasis on studies conducted in North America. To manage MDs like Parkinson's disease and Tourette syndrome, the results suggest that non-invasive neuromodulation techniques, closed-loop deep brain stimulation (DBS), and other advanced therapies may become the treatment of choice in the future. Research on dystonia is being focused on improving treatment methods by investigating new areas of the brain that might be stimulated through neurosurgery and looking at gene therapy. Modern technological developments, such as non-invasive neuromodulation procedures and improved imaging, provide promising substitutes for traditional surgical approaches. This study highlights the need for continuous clinical trials for better outcomes, which is why research and development in this area must continue.

## Introduction and background

Hyperkinetic and hypokinetic diseases make up a long list of movement disorders (MDs). In 1930, the first surgical procedures, pallidotomy and thalamotomy, were performed to alleviate Parkinson’s disease (PD) and dystonia. Parkinson’s disease is a hyperkinetic disorder with the presence of other non-motor symptoms. Similarly, dystonia is a hyperkinetic MD in which uncontrolled muscle contractions lead to different clinical manifestations [1-3]. Although PD and dystonia symptoms were initially alleviated with medication therapies such as levodopa and botulinum toxin, correspondingly, they were not successful in maintaining long-term desired effects. Levodopa in PD resulted in fluctuations, dyskinesias, and refractory tremors [2]. At the same time, to date, there are no known successful restoring treatments for dystonia but alleviating therapies that range from non-invasive to invasive means, whose outcomes are clearly differentiated when evaluating long-term effects and life quality [4,5].

At the beginning of the 1990s, pallidotomy and thalamotomy were replaced by the now well-known deep brain stimulation (DBS), which obtained FDA approval to treat PD, dystonia, essential tremor, and obsessive-compulsive disorder [2,6]. Once DBS is implanted, it is able to reshape irregular neural patterns, showing symptomatic superiority when compared to the standard medical-only treatment [7, 8]. Deep brain stimulation has lastly gained interest in treating neuropsychiatric disorders, including an interesting one, Gilles de la Tourette syndrome (GTS), normally diagnosed in childhood when patients develop spastic motor and verbal tics [9]. Even if still not approved by the FDA, promising DBS interventions have effectively taken place on selected patients, possibly overcoming the outcomes of conventional pharmacological and behavior/habit reversal therapies [5]. There is a lack of attention on similar multiple sclerosis (MS) and their shared available treatments. For instance, evaluating the pathophysiology of PD, dystonia, and GTS and the evolution of their treatments will permit us to find potential relationships among them, comprehend current treatment limitations, and use them as feedback for prospective solutions.

## Review

Parkinson’s disease

**Figure 1:**
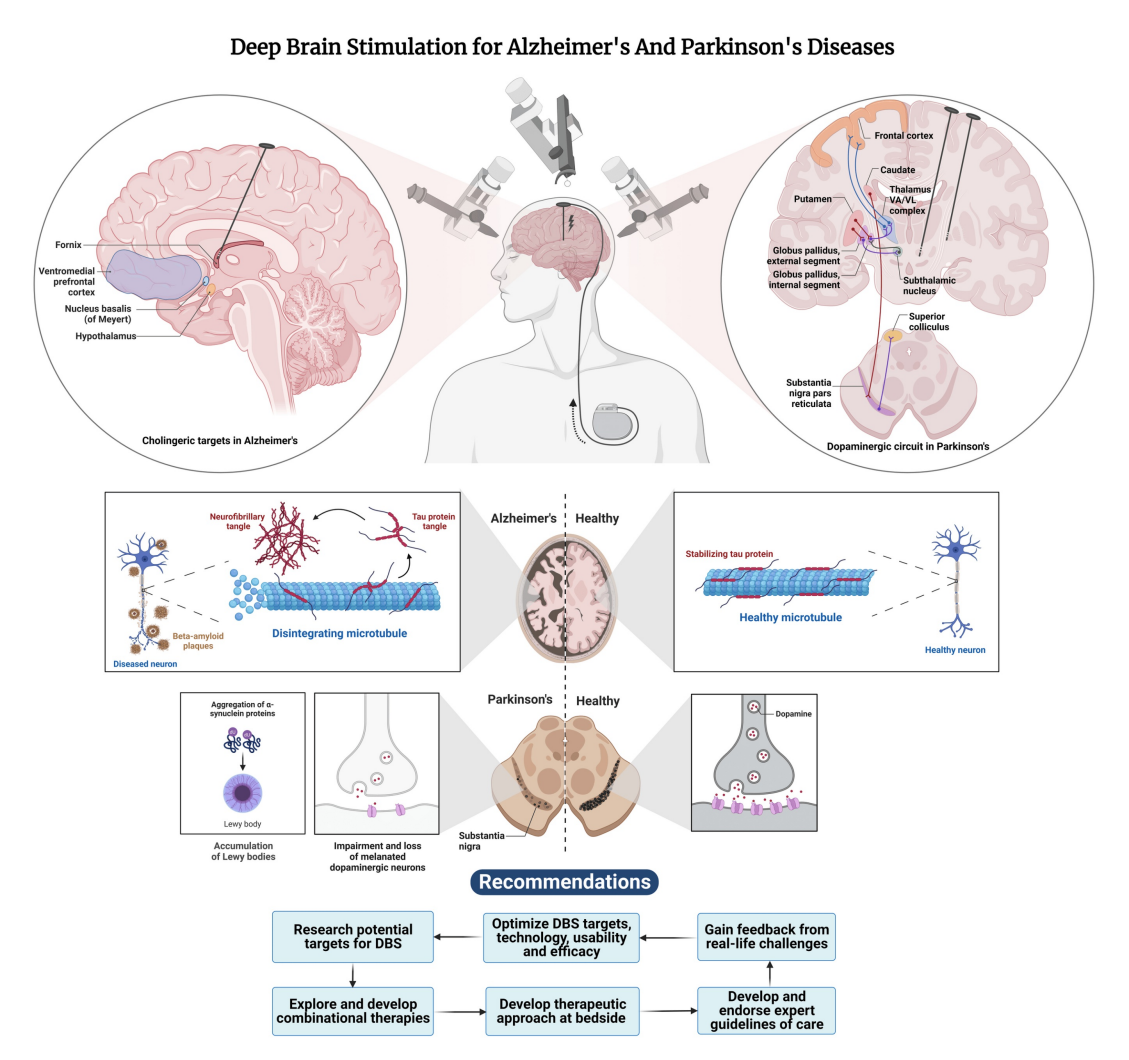
Deep brain stimulation of Parkinson’s and Alzheimer’s disease Reproduced under the terms and conditions of the Creative Commons Attribution (CC BY) license (https://creativecommons.org/licenses/by/4.0/) from reference [16]. Copyright © 2023 by the authors. Licensee MDPI, Basel, Switzerland.

Parkinson's disease is a common neurodegenerative disorder, presenting with tremors, bradykinesia/akinesia, muscular rigidity, abnormal gait, and cognitive impairment. This arises due to dopamine depletion in the basal ganglia [2]. The surgical management for Parkinson’s disease has metamorphosed over the past few decades, transitioning from stereotactic ablative surgeries to the current practice of DBS, focused ultrasound (FUS), neurostimulation, etc. Of these, the most thoroughly researched and commonly practiced technique is that of DBS [10,11]. Deep brain stimulation is generally used in medication-refractory PD tremors or in cases of intolerable side effects, motor fluctuations, and dyskinesias caused by conventionally utilized medications [12]. Deep brain stimulation has consistently proven to offer more benefits compared to lesioning procedures for the surgical management of movement disorders, especially PD. This is due to the vast number of advantages DBS provides, such as significantly improving motor functions, minimizing side effects, simplifying post-surgery pharmacological therapy, and the ability to program DBS, i.e., its adjustability (Figure 1) [7,13-15].

Mechanism of Action

Deep brain stimulation includes the surgical implantation of stimulation leads in various motor areas of the cortico-basal ganglia-thalamo-cortical circuit of the brain, including the subthalamic nucleus (STN), globus pallidus internus (GPi), and ventral intermediate nucleus (ViM) [17]. Recently, studies have also uncovered other sites for DBS, such as the pedunculopontine nucleus (PPN), to control PD-related tremors [18]. The above leads/electrodes are connected to internal pulse generators (IPGs), which are usually inserted in the subclavicular region, to modulate the lead signals. The motor area targeted and the programming of the IPG create a huge variation in the outcome of DBS, as discussed later. Some studies hypothesize that the target pathological activity is suppressed by the high-frequency electrical oscillations of DBS directed at the STN or GPi regions, ultimately resolving or reducing the debilitating clinical features of PD [14]. By disrupting the activity in these modulator motor pathways while retaining the normal information flow pathways, DBS has proved its efficacy in PD to successfully control tremors, rigidity, and bradykinesia [19, 20].

Indications for DBS IN PD

There are primarily three major indications for DBS to be employed in the management of PD. The cornerstone indication is for the cases of medication-refractory tremor. The second important indication for DBS includes patients facing the complications of chronic levodopa therapy like levodopa-induced dyskinesias and the wearing-off phenomenon caused by it. Thirdly, it is also an excellent option for some patients who may be intolerant to dopaminergic agents like levodopa [20,12].

Variation in Clinical Outcomes Based on Target Selection: STN vs. GPi DBS

Subthalamic nucleus and GPi are the most common targets for DBS. Some studies claim that STN DBS is typically used [21], whereas others state that GPi DBS is more commonly employed. Several studies have intended to differentiate the clinical outcomes of DBS on these sites [22]. The Unified Parkinson’s Disease Rating Scale (UPDRS) is widely regarded as the gold standard for measuring the severity and progression of PD. Many researchers use the UPDRS to compare and contrast the outcomes of STN versus GPi DBS. Many papers reveal that both STN DBS and GPi DBS have a similar outcome on tremor reduction, gait, and adverse effects such as changes in mood or apathy [21]. There is evidence that STN DBS is more effective than GPi DBS in terms of the potential for a larger reduction in the dosage of medication required by patients, consequently reducing the adverse effects associated with such dopaminergic medications, like hallucinations, drowsiness, compulsion behaviors, and orthostatic hypotension [12]. On the contrary, some information points towards GPi DBS being preferred over STN DBS when the concern is that of posture and stability [23]. Globus pallidus internus DBS may also be better than STN DBS to reduce levodopa-induced dyskinesia [12] and to improve balance [24]. Initially, STN DBP was speculated to be more associated with cognitive decline compared to GPi DBS. However, a review discovered only two out of nine randomized trials agreed with this conclusion. This provides substantial evidence to not completely exclude STN DBS as an effective form of treatment [25]. Furthermore, another study inferred that DBS on either site reduced tremor symptoms to a similar extent. However, the effectiveness of STN DBS versus GPi DBS on tremor symptoms varied in terms of fluctuation with time. Globus pallidus internus DBS showed a more uniform level of reduction in tremor symptoms over time, whereas post-STN DBS resulted in significant improvement in tremor symptoms at 24 and 60 months compared to six months following implantation [26]. Several neuropsychologists have believed that STN DBS is less safe than GPi DBS regarding neurobehavioral changes. However, a comprehensive review proves that the difference is not significant [27].

Improving clinical outcomes of DBS

Attempts are being made to improve the clinical outcomes of DBS, mainly with regard to multimodal neuroimaging [26, 28]. One such advancement is interventional magnetic resonance (iMRI)-guided DBS. In this technique, the STN/GPi electrode placement is done via intraoperative MRI guidance instead of only preoperative scans and placement of leads with the help of intraoperative symptom testing. Thus, when compared to traditional awake procedures, iMRI-guided DBS guarantees anatomically accurate placement of electrodes. This may help reduce the adverse effects of DBS associated with damage to neighboring structures during electrode placement. However, studies have found that the clinical outcomes of iMRI-guided DBS are akin to the outcomes of traditional DBS. Interventional magnetic resonance-guided DBS also offers no increased complications, risks, or operative times, despite initial hopes for an improved method [29]. Another effort directed toward improving the efficacy and minimizing the adverse effects of DBS in PD patients is adaptive DBS. It is a form of DBS that adjusts stimulation based on real-time feedback from brain activity rather than employing constant stimulation, which is seen in traditional DBS. Adaptive DBS is found to have similar therapeutic efficacy as conventionally used DBS. It has the potential to deliver more targeted stimulation and potentially reduce side effects [30-32]. Deep brain stimulation programming is another novel approach to improve the therapeutic effectiveness of DBS in PD, as discussed later.

Safety and adverse effects

Deep brain stimulation is not a risk-free intervention. Few complications occur following the surgical procedure, which may be associated with the implanted device or the procedure itself. Infection is the most prevalent surgery-related complication, while intracranial hemorrhage with potential permanent mental deficits is a common immediate complication associated with DBS. Weight gain is the most typical side effect associated with DBS seen in the post-surgery recovery period. Nausea, confusion, delirium, cognitive decline, pneumocephalus, and even mortality have been noticed as complications of DBS [1,7,22]. An important functional neurological complication following the procedure is speech and language impairment, including reduced verbal fluency, dysarthria, hypophonia, and ultimately, communication dissatisfaction [22,25,27,33]. Reduction in verbal fluency is a surgical implantation effect [34]. Memory, attention, and concentration may also be impacted [25]. Additionally, involuntary eye movement, muscle contraction, and postural instability are examples of neuromuscular complications that may occur due to damage to neighboring structures near the DBS target sites [33]. Multiple studies reveal that following bilateral (B/L) STN DBS, patients experience various mood changes. The sudden tapering of dopaminergic medication after B/L STN DBS results in apathy, anxiety, depression, suicidal attempts, and behavioral issues, with apathy being the most widely observed neuropsychiatric effect. Subthalamic nucleus DBS exacerbates apathy, which is a common symptom of PD itself [35]. However, these neuropsychological manifestations are relatively rare [27].

Quality of life (QoL) following DBS

Quality of life following DBS depends on several factors, such as the presence of multivariate cognitive decline, level of motor control regained, communication status, and neuropsychiatric effects developed [27]. Thus, the type of DBS is an important determinant in the future QoL for the patient. For example, some evidence shows that unilateral STN DBS is useful in improving depression six months postoperatively. This improvement in depression is typically sustained over time, leading to better sleep quality and overall QoL [36]. Contrarily, QoL is drastically reduced when complications of STN DBS, particularly apathy, arise [35]. Additionally, clinical outcomes may be unsatisfactory at times, even without any of the above-mentioned complications, regardless of the quality of the surgical procedure performed. In such cases, attempts are made to perform rescue procedures to improve quality of life [5]. However, research on such rescue procedures is inadequate and challenging to extrapolate to the general population. Therefore, in cases of suboptimal DBS results, QoL may deteriorate, and the risk of hospitalization may increase due to disease progression, which can be accelerated by other possible surgical complications [1].

Deep brain stimulation programming

**Figure 2:**
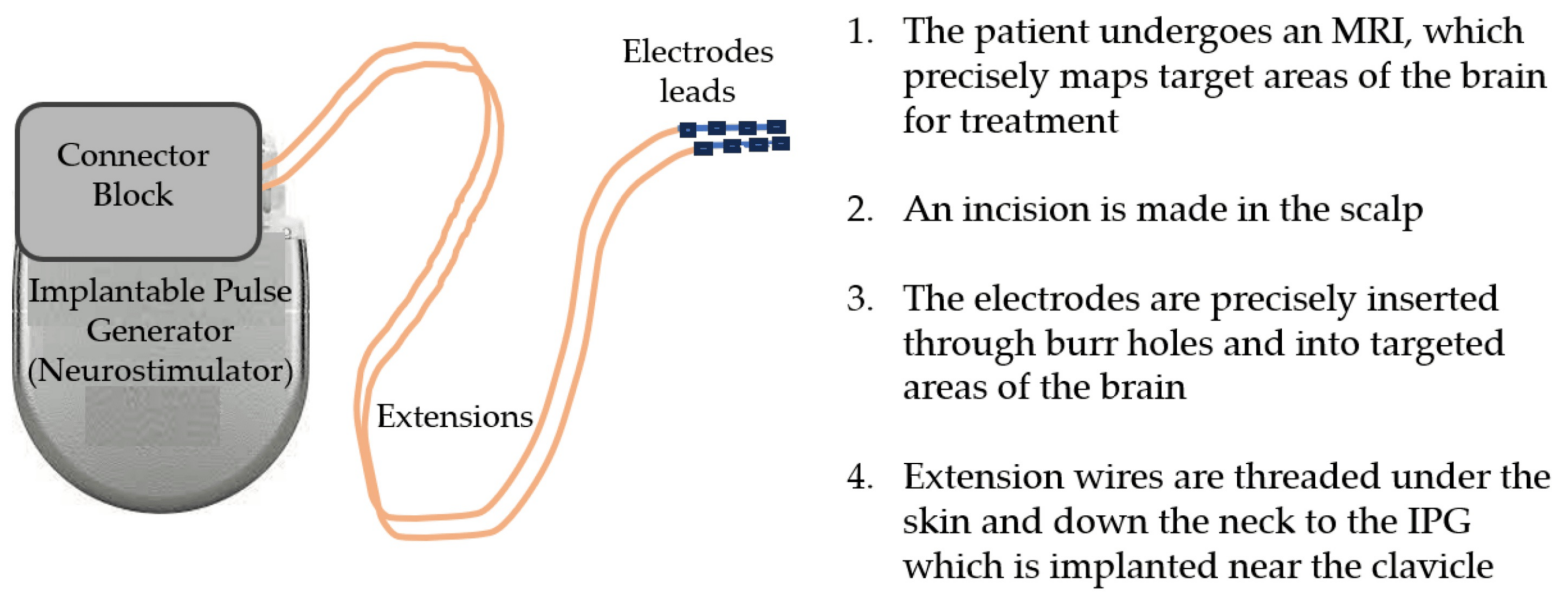
The deep brain stimulator system comprises the implantable pulse generator (IPG), the extensions that connect the IPG to the leads, and the electrode leads that are implanted into the brain. Reproduced under the terms and conditions of the Creative Commons Attribution (CC BY) license (https://creativecommons.org/licenses/by/4.0/) from ref. [38]. Copyright © 2023 by the authors. Licensee MDPI, Basel, Switzerland.

First DBS human systems owe their development to implants and battery design of cardiac pacemakers. Early applications of stimulators were for the treatment of pain by stimulation of the spinal cord; then, stimulation of the sensory thalamus arrived based on spinal cord stimulation (SCS) hardware technology. At this moment, DBS technology consisted of a single electrode with a single extension wire that was powered by a radio-frequency receiver and a device that was external and consisted of a transmitter driven by a battery of 9 volts. This transmitter was carried by the patient (Figure 2) [37].

By the 1970s, DBS was introduced for the treatment of movement disorders as a complement and eventually replacement of thalamotomies that were widely practiced by that time. First, for the treatment of MS-induced tremor, a primitive DBS system was developed with features similar to closed-loop systems and was only necessary for the activation of deltoid muscle by the patient to trigger stimulation of thalamic and subthalamic areas in order to stop the tremor [37].

Newer DBS systems came out in 1987 when Benabid et al. reported successful results managing tremor in patients with PD and ET using a DBS system with only one electrode contact placed at the ventromedial intermediate nucleus of the thalamus despite quadripolar electrodes (the current choice for electrodes) being available since the 1970s, and they induced chronic stimulation using radiofrequency (RF) coupled coils [39].

Referring to implantable pulse generators for DBS, the first one was developed by Medtronic, and its maximal frequency was 130 Hz (the current DBS value used in most of its applications), but it was only used for unilateral stimulation until 1999. This year, the first dual channel stimulator came out in Europe, a device that can deliver up to 250 Hz current, a feature that is especially helpful with the increasing use of bilateral DBS, especially for the subthalamic nucleus in the management of Parkinson's disease, characteristics that allowed this kind of IPG to become the most worldwide used. Then, the Activa series, the upcoming DBS hardware, came out with an expanded parameter space that clinicians could program, like the delivery of either a constant current or a constant voltage, and the possibility of programming different stimulation programs in an interleaving pattern. An inconvenience was the shorter battery life observed in these new devices and the need for replacement every three to four years, reasons that allowed the advent of rechargeable devices [37].

The major components of DBS devices are an internal system that includes a lead, electrodes, extension wires, and an implantable pulse generator, and an external system that consists of the clinician programmer, the patient, and, in some cases, a recharger [40]. The lead consists of a network of electrodes that are placed with stereotaxic surgery into a specific target inside the brain and then are attached to the IPG by extension wires; the IPG is often located subclavicularly, in the anterior wall of the chest or abdomen, depending on the patient's preferences [41].

Programming of DBS is essential to achieve accurate control and customization of this therapeutic in the management of movement disorders. It consists of the modification of electrode configuration and electrical parameters. The main parameters that can be programmed in DBS are the designation of a cathode, an anode, amplitude, pulse width, and rate [42].

Electrodes

The main aim of DBS is to deliver electrical impulses to specific brain targets using electrodes placed near these objectives. To select an adequate electrode, we must consider some features like biocompatibility, durability, inertness, surgical feasibility, tractability, stability over time, imaging compatibility, and electrical properties like conductivity. Current DBS electrodes are made of platinum-iridium cables and nickel alloy connectors inside a polyurethane sheath. Standard electrodes are configured as quadripolar, which means there are four stimulating contact points at the tip of the probe, with a 1.5-3mm length of each one, a parameter that is directly related to the range of neural targets and inversely related to its accuracy; contacts are spaced by 0-5-1.5mm of distance; and the probe has a diameter of 1.27-1.36mm [37].

There is a relevant concept that is necessary to talk about. The volume of tissue activated (VTA) refers to the amount of cerebral tissue that might be stimulated, serves as a gross representation of the powered electric field, and allows for predictions of the behavior of the nearby cerebral tissue, although the real number of stimulated neurons relies on several factors like distance from the electrode, myelination, and orientation of the fiber, etc. [41]. The VTA depends either on the number of total contact points, their polarities, or the characteristics of the surrounding tissue. Standard electrode placement allows for the shaping of VTA across the Z-axis of the lead, resulting in a symmetrical and omnidirectional isotropic supposed VTA; however, biophysical properties from brain tissue demonstrate that it has anisotropic properties, therefore asymmetric conductivities, and electric field gradients. Modeling of VTA is important in order to delimitate the therapeutic area and minimize risks for side effects induced by DBS. It's necessary to sculpt the VTA along the X and Y axes, not only in the Z axis, a necessity solved by segmented leads, which allow the development of directional DBS and the achievement of more accuracy targeting the desired regions and the capacity to steer the current that can improve the therapeutic window, widening the threshold before inducing side effects. Also, directional leads optimize therapeutic benefits throughout customized parameters due to the finding of specific areas that cause motor or non-motor clinical benefit [33]. A prospective double-blind randomized trial demonstrated directional stimulation with directional leads, compared to standard ring leads, achieved higher therapeutic windows (average increase of 41%), and the choice of directional contacts can help to reduce programmed amplitude for symptom relief or to increase the threshold for side effects, although the motor improvement was similar in both groups [42].

When a determined quantity of current is delivered to smaller contacts, a bigger density of current is generated; therefore, the threshold for amplitude stimulation is lower in segmented leads than in ring-shaped ones in order to avoid current densities that can cause irreversible damage to adjacent tissue. Deep brain stimulation programming of segmented leads is increasingly complex, and a possible solution for these issues could be automated programming, but further work is necessary to determine if automated programming can lead to equal or increased clinical benefit as observed with traditional DBS programming. Although segmented leads provide enhanced capabilities due to a larger number of contacts, they add surgical complexity to their implantation.

Biocompatibility of the electrodes is important due to the constant change in the interface created between electrodes and brain tissue; the electrical features of this interface are determined by glial encapsulation, protein adsorption, and ionic environment features. A general problem is an inflammatory response against a foreign body that needs to be counteracted if stable therapeutic responses are achieved. It's been demonstrated that giant multinucleate cells' response occurs independently of the duration of the implantation, probably against a component present within the surface coating of the electrodes like polyurethane; nevertheless, global experience suggests its long-term safety [37].

Implantable Pulse Generator and Programming

As we mentioned earlier, the first IPGs were only single-channel devices and could only accommodate one lead; then double-channel devices were introduced and nowadays are the most used due to the necessity of only one IPG for bilateral DBS leads. Battery life is a crucial feature of IPGs, and it's determined by several stimulation parameters, like monopolar or bipolar stimulation, frequency, amplitude, pulse width, battery chemistry, or even tissue parameters like variances of impedance [26].

The IPGs whose battery life is nearing its end tend to deliver lower current outputs than needed, causing rebounds of motor symptoms previously controlled. Unfortunately, there currently isn't an accurate model to predict the end of battery life, and sometimes there's a loss in therapeutic efficacy until IPG can be surgically removed [37].

The need for devices that can deliver high currents, avoiding depletion of battery life, has led to the coming out of rechargeable devices. These devices improve cost savings due to their less need for surgical replacement (their mean useful life is approximately 15 years) [41].

Stimulation Waveforms

Stimulation waveform is the trace that draws the voltage or current applied during a determined time and determines the extent, number, and type of brain cellular elements that can be stimulated; then these waves can be repeated at determined intervals of time in order to create a pattern of stimulation. It has been seen that symmetric biphasic pulses help more with motor symptoms in PD than asymmetric DBS waveforms, and these pulses are capable of activating more neurons at any intensity, probably due to both the participation of cathodic (current flowing out from the neuron and toward the electrode) and anodic (current flowing out of the electrode toward the neuron) phases to neurons activation. Other than symmetric and asymmetric waveforms, other elements also have to be taken into account, like interphase delays, reversal of the standard order of cathodic-anodic phases, bursts, and regularity or irregularities in time intervals [43].

Initiation of Programming

Deep brain stimulation programming doesn't begin immediately after electrode placement due to the possibility of the presence of microlesion effects; instead, programming is delayed two to four weeks after surgery, but it's completely done after a period of three to six months. The delay of beginning DBS programming occurs due to the induction of temporary clinical improvements due to the microlesion effect and possible confusion of the initial clinical outcomes following DBS and impedance changes that occur immediately after electrode placement due to edema but tend to stabilize during the next few weeks [26].

Stimulation Parameters

The parameters that determine the amount of stimulation delivered to the desired target inside the brain to go under DBS are the designation of the cathode, anode, amplitude, pulse width (PW), and rate of stimulation. All of these parameters shape the waveform, stimulation patterns, and therefore the VTA that can lead to different therapeutic effects.

Polarity

The polarity of electrodes can vary from monopolar stimulation, double, or triple monopolar stimulation, to bipolar or multiple stimulations. Monopolar stimulation is the standard setting where IPG is selected as the anode, and an electrode (or more) on the lead is designated as the cathode (or cathodes). On the other side, bipolar stimulation occurs when the cathode and anode are present on the electrodes on the lead. Monopolar stimulation creates a spherical field stimulation area and a larger VTA than bipolar stimulation; however, bipolar stimulation creates a constricted but elongated field, allowing a more accurate area of stimulation, diminishing probabilities for side effects, and requiring higher thresholds for the development of these [37].

Amplitude

Frequently is the first adjusted parameter during initial visits for DBS in PD, specifically during STN DBS [42]. Amplitude is directly related to VTA; therefore, if a larger amplitude is applied, then a larger number of neural elements will be stimulated, achieving a therapeutic effect but also running the risk of stimulating surrounding structures and increasing the risk of stimulation-side effects. Rigidity is the most important symptom to watch out for in PD due to its fast response and lack of fluctuations compared to tremors. [37].

Amplitude can be titrated by adjusting voltage or current, but newer IPGs use fixed currents regardless of the impedance of the system in order to diminish its variance with time. Although constant current devices are actually the most preferred by experts, more than one study reports no significant difference in clinical outcomes between using constant current or constant voltage. Tröster et al. found in a randomized controlled clinical trial assessing 136 PD patients who went under bilateral STN DBS suffered decreased letter fluency and Stroop's task test scoring, but depression improved a year after surgery [27].

Voltage-associated side effects consist of sensory or motor symptoms and are thought to be due to current spreading to the medial lemniscus and corticospinal tract, inducing paresthesias and muscle contractions, respectively. A trial that studied intraoperative clinical side effects produced by STN found that while using 130 Hz and 100 μs, the voltage threshold for the complete absence of wrist rigidity was 0.94V, and side effects began to appear at 2, 3, 3.1, and 3.4V for paresthesias, oculomotor, autonomous, and dystonic effects, respectively [44].

Frequency

The standard setting of frequency in DBS is 130 Hz; however, its adjustment can improve clinical outcomes and also diminish the chances of stimulation-induced side effects developing, as it’s been observed with low-frequency stimulation (under 100 Hz) that can improve freezing gait and axial rigidity in PD. A randomized, double-blinded crossover trial with a 14-patient sample compared low-frequency deep brain stimulation (LFS) (60 Hz) versus high-frequency deep brain stimulation (HFS) (130 Hz). It didn’t find significant differences in segmental signs between both groups, but it did find significant reductions in UPDRS-III, axial, and akinesia subscores, as well as a test of 10 meters walk in the group that went under 60 Hz DBS, and it was reported that the electrode placement for five patients was more ventrally than the usual for 130 Hz DBS [42]. Another observed benefit in symptoms using LFS was a 57% reduction of dysphagia and aspiration frequency in a randomized double-blind trial. Despite initial benefits in axial symptoms following LFS, it's been observed that these benefits might not be sustained over time [45]. Nevertheless, contrasting results are present, like occurred in the randomized trial performed by Phibbs et al., where they didn’t find any improvement in stride length or number of freezing episodes. Although two patients manifested significant improvement in their gate with no statistical significance in their outcomes, the authors also mention that these findings might occur due to the kept stimulation intensity at both frequencies, leading to lower electrical energy delivered during 60Hz frequency [23].

High-frequency stimulation, typically above 100 Hz, can improve tremor in patients with PD, but their immediate side effects have been widely reported, like worsening of gait or freezing. With these data, we might infer that LFS can help with axial symptoms and freezing of gait, and HFS can help manage tremors; however, patient variability must be considered, and, therefore, a wide range of frequencies during DBS should be performed during clinical practice [41].

Pulse Width

Standard PW can be divided into conventional PW DBS with PW >60μs and short pulse width DBS (<60μs), although it is usually set between 60-90 microseconds but technically can reach values up to 450μs; furthermore, newer IPGs can allow shorter values as 10μs. Reich et al. reported on the use of PW shorter than 60μs at a sustained frequency of 130 Hz in four patients that PW below 60μs can focus stimulation effect in smaller diameter axons closer to the leads [23,46], to avoid current spreading to corticospinal axons and therefore, motor side effects. They also found an inverse relationship between therapeutic windows, lower PW, and amplitude necessary to induce therapeutic effects; nevertheless, a charge per pulse required for tremor control decreased, proposing that shortening of PW might prolong the IPG's battery life, although more real-life study in battery life is needed [46].

A systematic review and meta-analysis that included 169 patients (143 with PD and 26 with essential tremor) found a wider therapeutic window with PW below 60μs but found no difference between therapeutic and side effects in various categories like non-motor symptoms, Movement Disorder Society (MDS)-UPDRS-III, quality of life, dyskinesia, gait, speech, in PD, but recommended it as optional in STN DBS; however, in patients with essential tremor, an increased tremor reduction was reported either [46].

Adaptive Stimulation and Closed-Loop Systems

An emerging form of programming that has caught a lot of attention lately is based on the idea of receiving feedback over time in the context of chronic brain stimulation. These technologies are able to modulate the stimulation in response to in vivo brain cellular changes and might help to prevent adverse effects. Closed-loop DBS differs from open-loop systems in that open-loop systems contain pre-programmed parameters that trigger stimulation patterns in the same way regardless of cellular changes or symptom status, while closed-loop systems send feedback based on neuronal biomarkers typical of the disease, during a very active status of symptoms or as a consequence of stimulation-induced side effects. There are also two types of closed-loop DBS systems: adaptive or responsive; whereas adaptive deep brain stimulation (aDBS) stimulation turns off after the symptomatic event "disappears," responsive deep brain stimulation (rDBS) stimulation persists after a fixed duration. Swann et al. demonstrated the feasibility of adaptive DBS in two patients with PD using a fully implanted neural prosthesis with a unilateral stimulation lead in the STN and a control paddle lead placed permanently subdural over the ipsilateral motor complex and found significant energy savings (38%-45%) maintaining therapeutic efficacy [32]. Feedback, a feature of adaptive DBS, needs a neural biomarker in order to work; hence, the recording of field potentials, specifically local field potential recordings, is in vivo and can provide insight into underlying neurophysiology and the mechanisms of DBS. Identification of these pathological biomarkers could help to decide the best site for placing the leads in order to achieve the maximal clinical benefit [30]. The possibilities of many biomarkers have been explored, highlighting beta oscillations due to their relationship with bradykinesia and rigidity, or gamma activity during dyskinesia in the motor cortex that is not affected by voluntary movement and has a larger amplitude as Swann et al. demonstrated.

Beta activity can be smoothed following drug therapy or present rapid fluctuations or prolonged, high-amplitude bursts of activity and be related to bradykinesia and rigidity and can work as a reliable biomarker to trigger DBS response [32]. In addition to these biomarkers, evoked potentials are also seen to appear in the order of milliseconds and are found both locally, subcortically, or remotely in the cortex, after DBS, and although they are promising as future biomarkers, there actually exist important gaps about their mechanism of appearance and implication in clinical settings [47].

There are specific chronic effects of the disease that have been seen to improve with STN DBS, like the sequence effect, which is defined as the progressive ongoing movement deterioration observed but not attributed to peripheral muscle fatigue, as was observed by Kehnemouyia et al., where they showed an improvement with open-loop systems and a direct relation between higher amplitudes of STN-DBS and improvement of the sequence effect, and also showed that the sequence effect is a condition that reflects progression of PD due to its worsening three years after off-therapy status and first programming [48].

Programming Visits

The initial visit for programming has a mean duration of 60-90 minutes, and it's a crucial visit to encourage the patient's family members or caregivers to accompany the patient in this first visit and educate the patient in order to achieve, in an easier manner, successful DBS programming and therefore, successful clinical outcomes. Patient education includes warning about possible side effects, how to turn on/off the stimulator, or how to adjust some parameters given to patients if it's possible or provided, as well as safety measures such as avoiding staying near strong magnetic fields or using diathermy during surgical procedures. Programming is typically performed during the morning under off-state medication to prevent overshadowing of pharmacological therapy above DBS results. This is achieved by asking the patient to hold overnight medications or to intentionally skip two doses in order to arrive in an off-medication status for the visit. After programming is done, the usual levodopa dose is administered to patients in order to determine optimal stimulation parameters to control dyskinesia induced by levodopa, which usually doesn't present after one dose until two or three doses are accumulated, and it's more frequent to observe during the afternoon. During the first six months after DBS surgery, patients following occur every month, and after adequate programming is achieved, they receive an annual basis for clinical performance, battery life, and side effects development [26].

Magnetic resonance-guided focused ultrasound (MRgFUS)

A recently emerging approach for the treatment of PD relies on focused ultrasound, an incision-less, ablative technique that had its beginnings proving its safety in animal models like rabbits or other primates [49]. Also, it was necessary to perform craniotomies in order to avoid wave deviations by the skull or trajectory-induced damage, and effective monitoring techniques were used [40]. Nowadays, it's a completely transcranial procedure guided by imaging, namely, MRgFUS, and consists of a helmet with approximately 1,000 individual piezoelectric transducers. These transducers deliver an epicenter of focused ultrasonic mechanic energy to a small brain tissue target that is absorbed and transformed into heat; significant heating is avoided by dispersing energy over the large cranial surface and by running degassed cold water that between the helmet and skull surface in order to cool the scalp. The array of transducers is connected to software that corrects differences in cranial bone thickness or factors that can lead to wave distortion, such as acoustic impedance [50], and therefore allows the accurate delivery of transcranial ultrasonic mechanic energy to the targeted nucleus, forming small lesions. Before permanent lesions are created, low frequency and therefore low-temperature discharges of ultrasonic energy are delivered in focal tissular zones that serve as control for planned targeted sites. Once the target is clinically verified, the temperature is elevated until the ablative threshold is reached, resulting in a permanent lesion [51].

Patient Considerations

Patients with advanced PD who aren't suitable candidates or refuse to go under DBS can be considered to go under MRgFUS. Variability of skull thickness between patients must be considered, establishing a skull density ratio >0.4 to be considered as a candidate for this treatment. Irreversibility and lack of real-time adjustability of ablative therapies like this or radiofrequency ablation must be considered. Patients with implants from previous neurosurgeries or skull aberrancies might not be adequate candidates for FUS, as well as the possibility of patients that have MRI-incompatible implants [51].

Clinical Outcomes in Ventral Intermediate Nucleus, Globus Pallidus Internus and Subthalamic Nucleus

During a double-center, double-blind, sham-controlled, pilot randomized trial of 27 patients with tremor dominant PD, hand tremor, which was evaluated with the Clinical Rating Scale for Tremor A+B subscores for hand tremor, improved 62% from baseline after FUS thalamotomy. Adverse effects reported during FUS were classified into two categories: thalamotomy-related and MRI/US environment-related. The most common side effects related to thalamotomy were paresthesia in the fingers (39%), ataxia (35%), and oral paresthesia (27%). Paresthesia persisted for up to one year in 19% of patients and ataxia in 4% [51].

A multicenter open-labeled trial tested the patient safety and efficacy of unilateral FUS of the GPi in 20 patients with PD who had responsiveness to levodopa, levodopa-induced dyskinesia, markedly asymmetrical motor symptoms, and at least a 30% difference in motor scores of the Movement Disorders Society version of the United Parkinson's Disease Rating Scale between the on and off state of medication. Authors expected improvement with unilateral treatment. Total Unified Dyskinesia Rating Scale scores improved 59% at three months after the MRgFUS, and this benefit was sustained during the whole study, 12 months after beginning; motor symptoms evaluated by MDS-UPRDS III improved 44.5% 3 months after baseline and 45.2% at 12 months compared to baseline. Adverse neurological effects were generally mild and transient and included dysarthria (20%), fine motor deficits (10%), visual field deficits (5%), cognitive disturbances (5%), and facial weakness (5%) [52].

Subthalamotomy for asymmetrical PD with MRgFUS has been tried, but a higher rate of adverse effects has been reported and therefore creates concerns. Martínez-Fernández et al. performed a prospective, open-label, pilot study where 10 patients with markedly asymmetric PD, poor pharmacotherapy control, patients who refused to go under DBS, bilateral DBS wasn't indicated, and of borderline age to go under DBS were enrolled. They found an improvement of 53% in MDS-UPRDS at six months under off-medication status and 47% under on-medication status. Nevertheless, at the six-month follow-up, 38 events of side effects were reported; none of them were severe. Seven side effects were persistent at that moment, and three were linked to subthalamotomy: subjective speech disturbance and dyskinesia in the treated arm regardless of the medication state that resolved after the levodopa reduction dose (in one patient who was in an on-medication state) and almost spontaneously resolved by the six months in the patient who was off-medication status. Other transient adverse effects related to subthalamotomy included gait ataxia (60%), pin site head pain (60%), and elevated blood pressure during the procedure (50%). Moderate impulsivity (20%) and transient facial asymmetry (10%) were also reported [40].

Magnetic Resonance-Guided Focused Ultrasound of the Pallidothalamic Tract (PTT)

The PTT is the main outflow tract of STN and GPi; it includes ansa lenticularis and fasciculus lenticularis and therefore is a possible target for ablative therapies, such as MRgFUS, as was demonstrated by Magara et al. on a prospective, open-labeled trial involving 13 patients with chronic PD and resistance to pharmacotherapy. These patients went under unilateral MRgFUS of PTT. The first four patients had target temperatures between 52°C and 59°C and experienced a mean improvement in MDS-UPDRS of 7.6% and thermocoagulation of 83 mm^2^ visible two days after the procedure but not at three months on T2 images. The next nine patients received four to five repetitions of the final temperatures and experienced a mean reduction in MDS-UPDRS of 60.9%; nevertheless, double-larger thermocoagulations were observed (172 mm^2^) in comparison to the first four patients. The MRI lesions were still evident after three months [53].

Cost Effectiveness

A meta-analysis that involved the review of 109 articles that included 3,573 patients performed by Mahajan et al., compared the cost-effectiveness between radio-frequency ablation and DBS and also predicted the threshold that FUS must overcome in order to become the most cost-effective treatment. This meta-analysis found DBS as the most cost-effective treatment in 22 months follow-up and, based on treatment cost, estimated adverse effects rate of 16.2%, and utility values obtained from Ravikumar et al. predicted percent reductions thresholds in UPDRS-III-Off reductions of 15.5% and 32.8% in order to achieve cost-effectiveness over two and five years period respectively [49].

Radiofrequency ablation

All ablative therapies used in neurological pathologies have as their main mechanism of action the destruction of brain tissue in specific areas. Specifically, radiofrequency ablation has recently stopped being used and has been replaced by other methods. Interstitial radiofrequency ablation is a therapeutic technique that generates a lesion using heat due to friction of ionic oscillations of an insulated electrode, but it’s not at the tip, or site of the active electrode; then, it's placed intracranially and coupled to a radiofrequency generator. When the generator is activated, current flows within the circuit and between active and dispersive electrodes and induces charged ions to move at approximately 500,000 cycles per second; frictional heating generated within the tissue is the mechanism of tissue heating. The most warmed point is at the zone of greatest current density, close to the tip of the active electrode, and, therefore, it's the specific place where the heat-induced injury occurs [51].

Anatomic Targets of Radiofrequency Ablation

In addition, most of the adverse effects from radiofrequency thalamotomy are transient and secondary to the edema generated by the ablation. There are other permanent adverse effects such as gait abnormalities (ataxia), motor deficits like dysarthria, or sensory deficits. These effects may be attributable to bilateral thalamotomy, where dysarthria is the most common adverse effect; therefore, unilateral thalamotomy is preferred [51].

When radiofrequency ablation is generated at the level of the globus pallidus internus, which is another of the main anatomical therapeutic targets, it also generates improvement in motor symptoms such as tremors, rigidity, bradykinesia, gait, and balance.

The same systematic review mentioned above reports findings from a controlled study that reported that patients who received unilateral pallidotomy obtained a 32% improvement in symptoms at six-month follow-up. Both thalamotomy and pallidotomy carry a great risk of cognitive and motor damage, even more so if a bilateral procedure is chosen, in addition to the fact that pallidotomy has been more commonly associated with visual deficits. It has another successful therapeutic target in radiofrequency ablation: the subthalamic nucleus, specifically to treat motor symptoms, in a review of the literature, reports in more recent studies an improvement of 43% to 52% in the UPDRS-III evaluations, adverse effects associated with subthalamotomy such as contralateral dyskinesias and transient hemiballismus, but there is a lower risk of neurocognitive problems in comparison to pallidotomy [51].

However, ablative procedures such as subthalamotomy are not preferred because they carry a higher risk of medically resistant hemiballismus. Although both therapeutic targets had very similar incidences of adverse effects in unilateral procedures, with subthalamotomy, the incidence of movement complications, including hemiballismus and dyskinesia, was 10 times more frequent [49]. Radiofrequency ablation therapy has been discontinued due to specific considerations, from intraoperative risks such as intracerebral hemorrhage to effects because they are irreversible and are not adjustable. Other drawbacks of this type of therapy are the lower accuracy of the size and shape of the lesion, which leads to different adverse effects, some temporary and others permanent or persistent [3].

Although this type of therapy is used less frequently in developed countries or with higher health incomes, this therapeutic option remains a reasonable strategy in low-income countries that do not have other safer therapeutic techniques available [51].

Cost Effectiveness of DBS Versus Radiofrequency: Is Radiofrequency Truly Cheaper Than DBS?

In a meta-analysis that compares the interventions available as a treatment for PD, it was found that DBS is the current neurosurgical treatment of choice for PD, replacing radiofrequency ablation. Discussion of this article focused on which option was more profitable, and bilateral DBS was found to cause lower direct costs than unilateral or bilateral RF because DBS device costs are frequently covered by the hospital. Parkinson's disease generates an accumulated economic burden associated with the management of the increasing disability due to the disease itself and the adverse effects of the different therapies used for management. In addition, it was concluded that DBS was more cost-effective since in the long term it incurred lower costs unlike RF due to its superior safety profile, which generates efficacy even in the early stages of PD; this leads to less disability in the patient and thus to a smaller number of therapeutic interventions and lower long-term costs [49].

Neurosurgical neuromodulation in PD

Neuromodulation involves the application of electrical impulses to the central, peripheral, and autonomic nervous systems through the alteration of neural signals. At the forefront, DBS has become the most notable in the management of refractory or uncontrolled PD [14]. However, there are other forms of neurosurgical neuromodulation, including those mentioned above, and others including stereotactic radiosurgery (SRS), transcranial magnetic stimulation, laser interstitial thermal therapy (LITT), and spinal cord stimulation.

Ablative therapies like RF ablation and SRS remain in use in resource-poor areas due to lower costs and simpler requirements, although DBS and MR FUS have replaced those neuromodulation surgeries in technologically advanced settings [40]. Lesioning surgeries including SRS and LITT are considered less invasive as they do not require burr holes or intracranial probes. Research has identified benefits in thalamotomy for tremor-dominant PD and pallidotomy for medication-resistant motor variations. Subthalamotomy, however, is less frequently performed as there is a risk of hemiballismus [12].

Stereotactic radiosurgery is a noninvasive technique using computerized dosimetry planning and image-guided stereotaxy to deliver a single dose of ionizing radiation to a particular intracranial target. Devices used in SRS include Gamma Knife and linear accelerators. Gamma Knife thalamotomy has demonstrated effectiveness in improving motor function in PD patients, with radiation doses ranging from 120 to 180 Gy. Studies have shown significant improvements in tremor score and motor functions following unilateral Gamma Knife thalamotomy, which is considered a good option for those patients who are unable to undergo more invasive procedures. On the downside, effects do take three to six months to manifest. In addition, Gamma Knife pallidotomy has also been explored, albeit less frequently, due to the high risk of optic neuropathy and other complications [40].

Laser interstitial thermal therapy is also used for ablating intracranial tissues by utilizing a laser diffusing fiber within a coiling catheter, which provides a real-time thermal image to control ablation. Though this technique is extensively used in epilepsy and brain tumors, its use in PD, specifically for pallidotomy and thalamotomy, is limited to only some case reports [40].

Decisions surrounding the appropriate surgical treatment require a multi-disciplinary team (MDT) approach with careful considerations of patient symptoms, expectations, and risk-benefit ratios. While DBS is preferred where available due to adjustability and suitability for bilateral use, other neuromodulation procedures may be appropriate for patients who cannot tolerate DBS hardware, and this does not take away from QoL outcomes in PD patients when compared to DBS [12].

Limitations

There are some notable limitations surrounding the role of neurosurgical interventions in PD. Regarding patient selection, patients with advanced disease who do not respond to pharmacological therapies are the typical target, leaving those with milder disease or those responding to medication unsuitable. Those who are unfit for surgery with significant comorbid conditions and serious cognitive impairments are usually not ideal candidates.

Surgical interventions can be very invasive and hence pose surgical risks, including bleeding, infection, and stroke. Postoperative complications like hardware concerns, decline in cognition, mood changes, and other side effects can pose significant risks. Okun et al. discuss worsening mental health conditions like depression and anxiety post DBS. Nonmotor symptoms are not well studied as part of the role of surgical interventions in PD, with the focus mainly being on motor symptoms [54].

Long-term management of patients undergoing DBS involves regular follow-up to adjust device settings and manage any side effects or complications, which can be burdensome for both patients and healthcare systems. Additionally, DBS systems require battery replacements every few years, necessitating additional surgical procedures and associated risks. Current neurosurgical interventions primarily address symptom control and do not modify the underlying disease progression of Parkinson's disease. There remains a limited understanding of the long-term effects of these interventions on disease progression and overall survival.

Access to specialized neurosurgical procedures may be limited by geographical, economic, or institutional factors, and not all patients may have access to experienced surgical teams and facilities. The cost of neurosurgical interventions, including surgery, device implantation, and long-term management, can be substantial and may not be fully covered by insurance, posing financial burdens on patients and healthcare systems. Additionally, patients may experience significant anxiety before surgery and may require psychological support to adjust to the outcomes and ongoing management of the intervention.

Discussion

Future research on the role of neurosurgical interventions in PD encompasses several areas included in the discussion below based on the information provided above.

Enhancing current techniques to involve improving the precision and efficacy of DBS through the development of adaptive systems that can adjust stimulation parameters in real-time based on neural feedback can be done to particularly optimize DBS. Identifying new brain regions as potential targets for neuromodulation could offer relief for a broader range of symptoms, including non-motor symptoms.

Additionally, there is a need for a clear understanding of the mechanism of action. Further studies are required to clarify the exact neurobiological effects of neurosurgical interventions like DBS and ablative surgeries, focusing on changes in neural circuitry, neurotransmitter levels, and neuroplasticity. Longitudinal studies are also needed to understand the long-term impact of these interventions on disease progression and overall brain health.

Research into advanced imaging technologies may provide alternatives or adjuncts to traditional surgery, as some studies in this selection have identified improvements in target accuracy when using high-resolution MRI.

The majority of studies have targeted improving motor symptoms of PD; however, PD also involves nonmotor symptoms, as mentioned earlier. Expansion of research to include this is crucial to improving QoL.

Cost-effectiveness and accessibility are also crucial areas to consider. Conducting cost-benefit analyses will help determine the economic viability of the widespread implementation of neurosurgical interventions. Developing strategies to make personalized medicine is an emerging field where developing more refined criteria and biomarkers can help better identify patients most likely to benefit from neurosurgical interventions. Tailoring these treatments to individual patient profiles, including personalized programming of DBS devices, can enhance outcomes.

Dystonia is a movement disorder defined by sustained muscle contractions, frequently causing twisting and repetitive movements with abnormal postures [55]. There are many types, including isolated, combined, focal, generalized, segmental, and cervical types [56]. Isolated dystonia is purely dystonia with no other symptoms, while combined dystonia is present with other disorders such as Parkinson’s disease. Focal dystonia usually affects one part of the body in comparison to generalized dystonia, where it affects the majority of the body. As for segmental, it affects two or more adjacent parts, while cervical dystonia is muscle contractions of the neck causing painful twisting of the head. The cause of dystonia remains uncertain; however, it may be due to abnormal GABAergic and dopaminergic transmission causing widespread dysfunction involving the cortex, brainstem, and spinal cord [3].

Deep brain stimulation can restore motor function in patients with dystonia by modulating dysfunctional neural circuits [57]. Restoration of functionality may lead to improvement of quality of life by reducing symptoms and allowing patients to perform daily activities. It is FDA-approved and is usually favored over ablation for severe, medication-refractory dystonia.

Wichmann and DeLong (2016) found GPi DBS was effective for isolated, generalized, segmental, and cervical dystonia; however, the response time varied for some patients [2]. Bledsoe et al. found GPi was highly efficacious for generalized and segmental dystonia, showing significant improvement in motor symptoms and daily activities; it was able to identify as more predictable in specific genetic dystonia, e.g., TOR1A mutations [58].

Subthalamic nucleus deep brain stimulation was found to be useful only in pilot studies, resulting in faster improvement. However, thalamic DBS was less commonly used for focal dystonia, while GPi and STN DBS were found to be more successful.

Elkaim et al. (2018) found in the pediatric population that patients with inherited dystonia without nervous system pathology (e.g., DYT1, DYT6, and myoclonus-dystonia) and idiopathic dystonia have a positive response to DBS; it shows robust responses with motor improvements ranging from 40.5% to 88.3% [59]. Poorer treatment response was linked to inherited dystonia with nervous system dysfunction, acquired dystonia, younger age of onset, and absence of involvement of the trunk [60].

Potential risks associated with DBS include intracerebral hemorrhages, cerebrospinal fluid leaking, seizures, infections, and complications connected to the hardware used [61]. Side effects may be managed by adjusting stimulation parameters.

Globus pallidus internus lesioning was initially used for levodopa-induced dyskinesia and was superseded by DBS due to reversibility, better tolerability, and fewer side effects.

The most recent non-invasive lesioning method is MRgFU, which is used mostly in focal hand dystonia, targeting the ventralis intermedius thalamus [18]. Clinical outcomes consistently showed improvement post treatment, with statistically significant enhancements in dystonia and tremor measures reported in some studies. However, there were adverse effects, such as transient sensorimotor issues.

Rhizotomy, ablation of the peripheral nerves, can be used for hypertonia, especially for segmental or limb-specific hypertonia, e.g., torticollis. It is considered a palliative rather than a therapeutic treatment, as it may not address the underlying etiology of dystonia [62].

## Conclusions

In conclusion, neurosurgical interventions, particularly DBS, have demonstrated significant benefits in treating movement disorders like PD, dystonia, and Tourette syndrome. Advancements in technology, such as adaptive DBS and MRI-guided procedures, enhance the precision and effectiveness of these treatments, offering better symptom management and improved quality of life. Despite the challenges and risks, ongoing research into non-invasive techniques, new brain targets, and personalized therapies continues to expand treatment possibilities. Future efforts should focus on optimizing safety, efficacy, and accessibility, ensuring that more patients can benefit from these life-changing interventions.

## References

[1] Olson MC, Shill H, Ponce F, Aslam S (2023). Deep brain stimulation in PD: risk of complications, morbidity, and hospitalizations: a systematic review. Front Aging Neurosci.

[2] Wichmann T, DeLong MR (2016). Deep brain stimulation for movement disorders of basal ganglia origin: restoring function or functionality?. Neurotherapeutics.

[3] Jinnah HA, Alterman R, Klein C, Krauss JK, Moro E, Vidailhet M, Raike R (2017). Deep brain stimulation for dystonia: a novel perspective on the value of genetic testing. J Neural Transm (Vienna).

[4] Bohn E, Goren K, Switzer L, Falck-Ytter Y, Fehlings D (2021). Pharmacological and neurosurgical interventions for individuals with cerebral palsy and dystonia: a systematic review update and meta-analysis. Dev Med Child Neurol.

[5] Johnson KA, Worbe Y, Foote KD, Butson CR, Gunduz A, Okun MS (2023). Tourette syndrome: clinical features, pathophysiology, and treatment. Lancet Neurol.

[6] Nagy AM, Tolleson CM (2016). Rescue procedures after suboptimal deep brain stimulation outcomes in common movement disorders. Brain Sci.

[7] Hacker ML, Turchan M, Heusinkveld LE (2020). Deep brain stimulation in early-stage Parkinson disease: five-year outcomes. Neurology.

[8] Sanger TD (2020). Deep brain stimulation for cerebral palsy: where are we now?. Dev Med Child Neurol.

[9] Müller-Vahl KR, Szejko N, Saryyeva A (2021). Randomized double-blind sham-controlled trial of thalamic versus GPi stimulation in patients with severe medically refractory Gilles de la Tourette syndrome. Brain Stimul.

[10] Ahmed H, Field W, Hayes MT, Lopez WO, McDannold N, Mukundan S Jr, Tierney TS (2015). Evolution of movement disorders surgery leading to contemporary focused ultrasound therapy for tremor. Magn Reson Imaging Clin N Am.

[11] Sun FT, Morrell MJ (2014). Closed-loop neurostimulation: the clinical experience. Neurotherapeutics.

[12] Sharma VD, Patel M, Miocinovic S (2020). Surgical treatment of Parkinson's disease: devices and lesion approaches. Neurotherapeutics.

[13] Picillo M, Phokaewvarangkul O, Poon YY (2021). Levodopa versus dopamine agonist after subthalamic stimulation in Parkinson's disease. Mov Disord.

[14] Cole RC, Okine DN, Yeager BE, Narayanan NS (2022). Neuromodulation of cognition in Parkinson's disease. Prog Brain Res.

[15] Hickey P, Stacy M (2016). Deep brain stimulation: a paradigm shifting approach to treat Parkinson's disease. Front Neurosci.

[16] Senevirathne DK, Mahboob A, Zhai K (2023). Deep brain stimulation beyond the clinic: navigating the future of Parkinson’s and Alzheimer’s disease therapy. Cells.

[17] Loh A, Gwun D, Chow CT (2022). Probing responses to deep brain stimulation with functional magnetic resonance imaging. Brain Stimul.

[18] Hamani C, Lozano AM, Mazzone PA (2016). Pedunculopontine nucleus region deep brain stimulation in Parkinson disease: surgical techniques, side effects, and postoperative imaging. Stereotact Funct Neurosurg.

[19] Chandra V, Hilliard JD, Foote KD (2022). Deep brain stimulation for the treatment of tremor. J Neurol Sci.

[20] Merola A, Singh J, Reeves K (2021). New frontiers for deep brain stimulation: directionality, sensing technologies, remote programming, robotic stereotactic assistance, asleep procedures, and connectomics. Front Neurol.

[21] Okun MS, Wu SS, Fayad S (2014). Acute and chronic mood and apathy outcomes from a randomized study of unilateral STN and GPi DBS. PLoS One.

[22] França C, Carra RB, Diniz JM, Munhoz RP, Cury RG (2022). Deep brain stimulation in Parkinson's disease: state of the art and future perspectives. Arq Neuropsiquiatr.

[23] Phibbs FT, Arbogast PG, Davis TL (2014). 60-Hz frequency effect on gait in Parkinson's disease with subthalamic nucleus deep brain stimulation. Neuromodulation.

[24] St George RJ, Carlson-Kuhta P, Nutt JG, Hogarth P, Burchiel KJ, Horak FB (2014). The effect of deep brain stimulation randomized by site on balance in Parkinson's disease. Mov Disord.

[25] Mehanna R, Bajwa JA, Fernandez H, Wagle Shukla AA (2017). Cognitive impact of deep brain stimulation on Parkinson’s disease patients. Parkinsons Dis.

[26] Wong JK, Cauraugh JH, Ho KW (2019). STN vs. GPi deep brain stimulation for tremor suppression in Parkinson disease: a systematic review and meta-analysis. Parkinsonism Relat Disord.

[27] Tröster AI (2017). Some clinically useful information that neuropsychology provides patients, carepartners, neurologists, and neurosurgeons about deep brain stimulation for Parkinson's disease. Arch Clin Neuropsychol.

[28] Peng S, Dhawan V, Eidelberg D, Ma Y (2021). Neuroimaging evaluation of deep brain stimulation in the treatment of representative neurodegenerative and neuropsychiatric disorders. Bioelectron Med.

[29] Lee PS, Richardson RM (2017). Interventional MRI-guided deep brain stimulation lead implantation. Neurosurg Clin N Am.

[30] Neumann WJ, Turner RS, Blankertz B, Mitchell T, Kühn AA, Richardson RM (2019). Toward electrophysiology-based intelligent adaptive deep brain stimulation for movement disorders. Neurotherapeutics.

[31] Little S, Brown P (2020). Debugging adaptive deep brain stimulation for Parkinson's disease. Mov Disord.

[32] Swann NC, de Hemptinne C, Thompson MC (2018). Adaptive deep brain stimulation for Parkinson's disease using motor cortex sensing. J Neural Eng.

[33] Schnitzler A, Mir P, Brodsky MA (2022). Directional deep brain stimulation for Parkinson's disease: results of an international crossover study with randomized, double-blind primary endpoint. Neuromodulation.

[34] Cernera S, Okun MS, Gunduz A (2019). A review of cognitive outcomes across movement disorder patients undergoing deep brain stimulation. Front Neurol.

[35] Vachez YM, Creed MC (2020). Deep brain stimulation of the subthalamic nucleus modulates reward-related behavior: a systematic review. Front Hum Neurosci.

[36] Birchall EL, Walker HC, Cutter G (2017). The effect of unilateral subthalamic nucleus deep brain stimulation on depression in Parkinson's disease. Brain Stimul.

[37] Krauss JK, Lipsman N, Aziz T (2021). Technology of deep brain stimulation: current status and future directions. Nat Rev Neurol.

[38] Whitestone J, Salih A, Goswami T (2023). Investigation of a deep brain stimulator (DBS) system. Bioengineering (Basel).

[39] Benabid AL, Pollak P, Louveau A, Henry S, de Rougemont J (1987). Combined (thalamotomy and stimulation) stereotactic surgery of the VIM thalamic nucleus for bilateral Parkinson disease. Appl Neurophysiol.

[40] Serva SN, Bernstein J, Thompson JA, Kern DS, Ojemann SG (2022). An update on advanced therapies for Parkinson's disease: from gene therapy to neuromodulation. Front Surg.

[41] Frey J, Cagle J, Johnson KA (2022). Past, present, and future of deep brain stimulation: hardware, software, imaging, physiology and novel approaches. Front Neurol.

[42] Dayal V, Limousin P, Foltynie T (2017). Subthalamic nucleus deep brain stimulation in Parkinson’s disease: the effect of varying stimulation parameters. J Parkinsons Dis.

[43] Akbar U, Raike RS, Hack N (2016). Randomized, blinded pilot testing of nonconventional stimulation patterns and shapes in Parkinson’s disease and essential tremor: evidence for further evaluating narrow and biphasic pulses. Neuromodulation.

[44] Sauleau P, Raoul S, Lallement F, Rivier I, Drapier S, Lajat Y, Verin M (2005). Motor and non motor effects during intraoperative subthalamic stimulation for Parkinson's disease. J Neurol.

[45] Xie T, Vigil J, MacCracken E (2015). Low-frequency stimulation of STN-DBS reduces aspiration and freezing of gait in patients with PD. Neurology.

[46] Smeets S, Boogers A, Van Bogaert T (2024). Deep brain stimulation with short versus conventional pulse width in Parkinson's disease and essential tremor: a systematic review and meta-analysis. Brain Stimul.

[47] Dale J, Schmidt SL, Mitchell K, Turner DA, Grill WM (2022). Evoked potentials generated by deep brain stimulation for Parkinson's disease. Brain Stimul.

[48] Kehnemouyi YM, Petrucci MN, Wilkins KB, Melbourne JA, Bronte-Stewart HM (2023). The sequence effect worsens over time in Parkinson’s disease and responds to open and closed-loop subthalamic nucleus deep brain stimulation. J Parkinsons Dis.

[49] Mahajan UV, Ravikumar VK, Kumar KK (2021). Bilateral deep brain stimulation is the procedure to beat for advanced Parkinson disease: a meta-analytic, cost-effective threshold analysis for focused ultrasound. Neurosurgery.

[50] Baek H, Lockwood D, Mason EJ (2022). Clinical intervention using focused ultrasound (FUS) stimulation of the brain in diverse neurological disorders. Front Neurol.

[51] Jankovic J, Cardoso F, Grossman RG, Hamilton WJ (1995). Outcome after stereotactic thalamotomy for parkinsonian, essential, and other types of tremor. Neurosurgery.

[52] Eisenberg HM, Krishna V, Elias WJ, Cosgrove GR, Gandhi D, Aldrich CE, Fishman PS (2021). MR-guided focused ultrasound pallidotomy for Parkinson's disease: safety and feasibility. J Neurosurg.

[53] Magara A, Bühler R, Moser D, Kowalski M, Pourtehrani P, Jeanmonod D (2014). First experience with MR-guided focused ultrasound in the treatment of Parkinson's disease. J Ther Ultrasound.

[54] Almeida L, Deeb W, Spears C (2017). Current practice and the future of deep brain stimulation therapy in Parkinson's disease. Semin Neurol.

[55] Dressler D, Kopp B, Pan L, Saberi FA (2024). The natural course of idiopathic cervical dystonia. J Neural Transm (Vienna).

[56] Stephen CD, Dy-Hollins M, Gusmao CM, Qahtani XA, Sharma N (2023). Dystonias: clinical recognition and the role of additional diagnostic testing. Semin Neurol.

[57] Balint B, Mencacci NE, Valente EM (2018). Dystonia. Nat Rev Dis Primers.

[58] Bledsoe IO, Viser AC, San Luciano M (2020). Treatment of dystonia: medications, neurotoxins, neuromodulation, and rehabilitation. Neurotherapeutics.

[59] Malatt C, Tagliati M (2022). Long-term outcomes of deep brain stimulation for pediatric dystonia. Pediatr Neurosurg.

[60] Hale AT, Monsour MA, Rolston JD, Naftel RP, Englot DJ (2020). Deep brain stimulation in pediatric dystonia: a systematic review. Neurosurg Rev.

[61] Fenoy AJ, Simpson RK Jr (2014). Risks of common complications in deep brain stimulation surgery: management and avoidance. J Neurosurg.

[62] McEvoy SD, Limbrick DD, Raskin JS (2023). Neurosurgical management of non-spastic movement disorders. Childs Nerv Syst.

